# Community participation in the retention of adults in HIV care in the Muyuka Health District, South-West Region, Cameroon

**DOI:** 10.11604/pamj.2019.33.32.17174

**Published:** 2019-05-16

**Authors:** Enongene-Kome Constance, Hermann Ngouakam, Egbe Obinchemti Thomas, Dickson Shey Nsagha

**Affiliations:** 1 Department of Public Health and Hygiene, Faculty of Health Sciences, University of Buea, Buea, Cameroon; 2 Department of Gynecology and Obstetrics, Faculty of Health Sciences, University of Buea, Buea, Cameroon

**Keywords:** Retention in HIV care, antiretroviral therapy, community participation

## Abstract

**Introduction:**

Antiretroviral therapy (ART) is vital for people living with HIV (PLWHIV) and a substantial number of HIV/AIDS patients still face stigmatization from family and other members of the community. Stigma could lead to poor retention in HIV care and consequently result in decreased chances of survival and increased risk of HIV transmission. The aim of this study was to determine the retention of patients in HIV care and community participation in the retention of patients in HIV care at the Muyuka Health District, South-West Region, Cameroon.

**Methods:**

This was an analytic cross-sectional retrospective study where 385 hospital records of people living with HIV (PLWHIV) enrolled in HIV care were reviewed, and we administered 348 questionnaires to community members. Data were collected and analysed using bivariate analysis and chi-square test. The Susan Rifkin's scoring method was used to measure community participation. Statistical significance was set at P-value < 0.05.

**Results:**

A total number of 112(29.1%) of people living with HIV (PLWHIV) enrolled in HIV care were retained in HIV care against 273(70.9%), who were lost to follow-up over a two year and four months period. Patients on a Zidovudine containing ART regimen were about 7 times more likely to be lost to follow-up (OR 6.92; 95% CI 1.80-26.60, P-value = 0.005). The overall community participation in the retention of adults in HIV care in the Muyuka Health District was low; mean resource allocation score = 2.43, mean leadership score = 1.0; mean organization factor score = 1.30; but the mean needs assessment score was good (4.0).

**Conclusion:**

retention of patients enrolled in HIV care, and the community participation in the retention were low. Collaborations between health care structures and community initiatives should be resourced to foster continuum of care for people living with HIV (PLWHIV).

## Introduction

Antiretroviral therapy (ART) is essential for Human Immuno-deficiency Virus (HIV) infected patients to ensure increased survival and decreased HIV transmission. Retention in care is the ability to adhere to critical aspects of care such as attending regular follow-up appointments, scheduled laboratory tests and other monitoring activities as prescribed by the health care provider [1]. Majority of the people living with HIV worldwide are in sub-Saharan Africa, with an estimated 25.5 million cases out of the 36.7 million cases recorded worldwide [2]. Among this group, 19.4 million are living in East and Southern Africa which saw 44% of new infections globally in 2016 [2]. As of July, 2017 out of the 36.7 million people living with HIV, an estimated 20.9 million people living with HIV (PLWHIV) were accessing ART globally implying that 15.8 million people living with HIV (PLWHIV) do not have access to antiretroviral therapy [2]. In consequence, 1 million people died from AIDS-related illness in 2016 [2]. In 2016, it was recorded that there are about 560,000 PLWHIV in Cameroon and only about 37% of these patients have access to ART [3]. Since the year 2000, an estimated 25 million deaths have been recorded among people living with HIV with most of these deaths observed in Sub-Saharan Africa [4]. Due to the HIV/AIDS related mortality, there have been tremendous global efforts from the global health community, leading governments and civil society organizations by scaling-up treatment for HIV patients [5]. The HIV pandemic does not only affect the health of individuals but has an impact on households, communities, and the development of economic growth of nations since majority of these countries, mostly affected by the pandemic, also suffer from other infectious diseases, food security and other serious problems [6]. A consensus was reached on the fact that communities should be actively involved in improving their own health [7]. Community participation in health programmes consists of an adequate response to the needs of the community, designing health programmes aimed at mitigating health problems that greatly affect the community, and foster the increase of public accountability of health problems [7, 8]. The growing HIV burden on families and health systems is exerting a shift toward community care-givers and increasing the demand for functional community systems [6]. In Cameroon, the HIV pandemic is against a weak health system where, the role of the community is poorly understood. It was therefore imperative to conduct a study on community participation in the retention of adults in HIV care in the Muyuka Health District (MHD).

## Methods

This was an analytic cross-sectional retrospective study, conducted at the Muyuka District Hospital in the Muyuka Health District. The Muyuka District Hospital is one of the primary health care facilities found in the South-West Region, Cameroon. This study was conducted in two sections: the first part comprised of the retrospective part of the study which reviewed hospital records of people living with HIV (PLWHIV) enrolled in HIV care at the HIV unit of the hospital to measure retention of HIV patients in HIV care. Records of patients enrolled in HIV care from the 1^st^ of January, 2016 to the 30^th^ of April, 2018 were reviewed. Patient records were reviewed for socio-demographic information, WHO stage, CD4 count, number of follow-up visits, and patient retention status (defined as client attending at least one clinic visit per year [9]). An aggregate of 385 records of people living with HIV (PLWHIV) enrolled in HIV care were reviewed. Patients who were below 21 years during the research period were excluded from the study. All patients who were tested positive but were not enrolled in HIV care were not considered to be retained in HIV care. Patient outcome in care were evaluated as “in care” or loss to follow-up. The second part of the study was a cross-sectional study, which entailed the administration of questionnaires to adult members of the Muyuka community to measure the level of community participation in the retention of adults in HIV care (community members were both people living with HIV (PLWHIV) receiving treatment at the HIV unit and other members of the community whose HIV status were unknown e.g. health care providers and family members). The Susan Rifkin's scoring method was used to measure the level of community participation. Four out of the five components of the Susan Rifkin's scoring method was measured in line with the scope of this study (needs assessment, organization, leadership and resource mobilization). The questionnaires used in this study were adapted from the European Social Survey (ESS) and it contained questions on needs assessment, leadership, organization and resource mobilization which assisted the researchers grade the level of community participation.

Needs assessment measured the degree to which members of the community could determine the needs of people living with HIV (PLWHIV) for continuous retention in HIV care. Organization evaluated the capability of community members to steer up or engage in community projects that support people living with HIV (PLWHIV) in HIV care. Resource mobilization determined how members of the community support people living with HIV (PLWHIV) enrolled in HIV care financially, and leadership assessed how leaders in the community represented the interests of the community and their ability to steer up community projects. All of these four components were measured on a scale of 1-5 (1 = very low, 2 = low, 3 = good, 4 = very good, and 5 = excellent). The questionnaires were pre-tested among community members of the Muea health area. A cumulative number of 348 pre-tested questionnaires were administered to adult members of the Muyuka community. Administration of study questionnaires was conducted following a multi-stage sampling technique. Participants were grouped into two strata which comprised people living with HIV (PLWHIV) receiving treatment at the HIV unit and other members of the community whose HIV status were unknown. A component of the study population comprised people living with HIV (PLWHIV) in order to grade their level of care (care is person-centered) which was an element of needs assessment criteria to measure community participation. Probability proportionate to size sampling was used to determine the total number of participants per strata. Participants from these two strata were randomly selected to partake in the study. Those who had only been residing in the Muyuka health area for less than six months were excluded from the study. Data were entered on Microsoft excel worksheet, 2013, daily. Data were analysed using SPSS version 20.0. Descriptive statistics was used to determine the overall retention of people living with HIV (PLWHIV) enrolled in HIV care and to measure the level of community participation. Chi-square test was used to determine the association between patient characteristics and appointment adherence. Ethical approval for this study was obtained from the Institutional Review Board, Faculty of Health Science, University of Buea (Ref: 2018/0218/UB/SG/IRB/FHS). The limitations of this study entailed that the retrospective nature of the study at its discretion reduce the statistical power and lead to biased estimates attributable to missing data from patient files and the cross-sectional nature of the study may not lead to the study of cause and effect.

## Results

**Figure 1:**
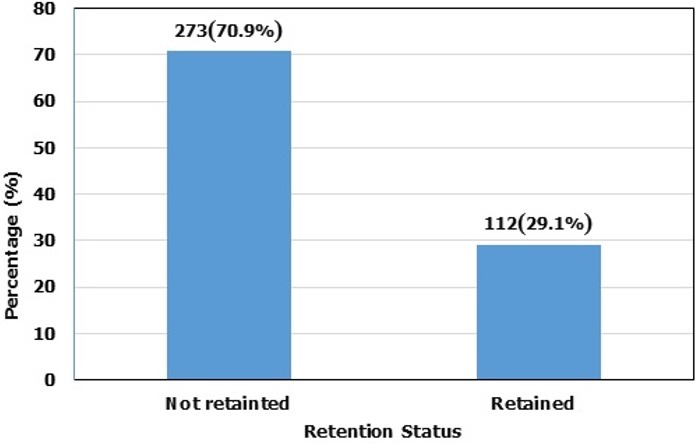
Retention status in HIV care over a two years and four-month period at the Unit in-charge of patients with HIV (UPEC) of the Muyuka District Hospital (January, 2016-April, 2018)

**Figure 2:**
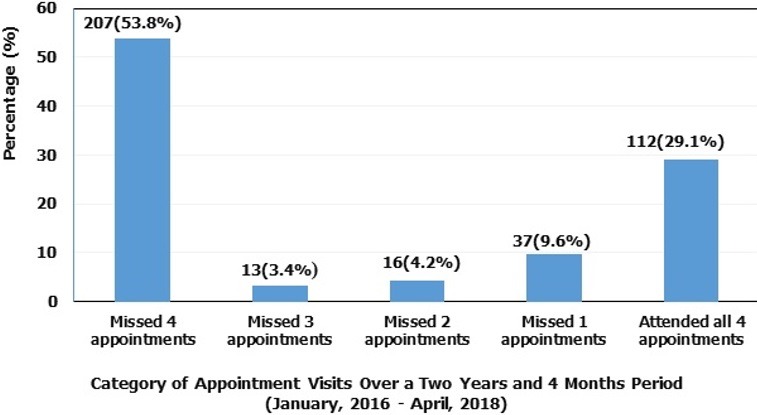
Distribution of appointment visits over a two years and four-month period at the UPEC of the Muyuka District Hospital (January, 2016-April, 2018)

**Figure 3:**
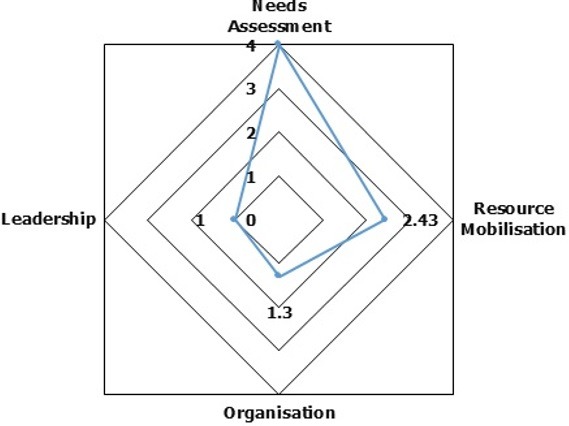
Overall level of community participation in the retention of adult HIV/AIDS patients in HIV care, April, 2018 at the Muyuka Health District

The study participants were females 284 (73.8%) and 101 (26.2%) were males (Table 1). The age group that was most common among the study participants was 31-40 years with over 43.6% of the participants falling in this category (Table 1). An aggregate of 175 (45.5%) of the study participants had CD4 lymphocyte test done prior to initiation to antiretroviral therapy. Forty percent (40%) of patients who carried out the CD4 lymphocyte test had a CD4 lymphocyte count of < 200cells/µl at initiation of therapy. According to WHO clinical staging, 44.6% of the study participants were at stage II and 3.3% were at stage IV at initiation of therapy. With regard to the ART regimen, most of the patients (97.1%) were on the Tenofovir containing regimen while those on the Zidovudine containing regimen were fewer in number (2.9%) prior to initiation to antiretroviral therapy (Table 1). Three hundred and forty-eight (348) questionnaires were administered to community members. Majority of the study participants were females: 213 (61.2%) and 135 (38.8%) were males (Table 2). The age group of 21-30 years were more common, accounting for more than half (54.9%) of the community participants. Most of the participants in this study were middle income earners, 118 (33.9%), single 179 (51.4%), and 318 (91.4%) were Christians (Table 2). One hundred and twelve (112) patients were retained in HIV care as opposed to 273 who were loss to follow-up. The overall retention rate of people living with HIV (PLWHIV) enrolled in HIV care was 29.1%. (Figure 1). About 53.8% of patients had 4 missed visits, 3.4% of the patients had 3 missed visits, 4.2% of the patients had 2 missed visits and 9.6% of the patients had 1 missed visit. (Figure 2). Respondents receiving the Zidovudine containing ART regimen were about 7 times more likely to be lost to follow-up (OR 6.92; 95% CI 1.80-26.60, P-value = 0.005), compared to those on a Tenofovir containing ART regimen (Table 3). The overall level of community participation of adults enrolled in HIV care was low. The mean score of the needs assessment factor was very good (mean score of approximately 4/5), the mean score of the leadership factor was very low (mean score of approximately 1/5). The mean score on the resource allocation factor was low (mean score of 2.43/5). In addition, the mean score on the organizational factor was very low (mean score of 1.3/5) (Figure 3).

**Table 1:** Baseline characteristics and pre-treatment clinical characteristics of study participants enrolled in HIV care, between January, 2016 to April, 2018 at the Muyuka District Hospital

Characteristic		Frequency	Percentage (%)
**Gender**			
	Male	101	26.2
	Female	284	73.8
	**Total**	**385**	**100.0**
**Age group (years)**			
	21-30	80	20.8
	31-40	168	43.6
	41-50	88	22.9
	51-60	35	9.1
	>60	14	33.6
	**Total**	**385**	**100.0**
**Weight category**			
	<50 kg	45	23.6
	50-70 kg	116	60.7
	>70 kg	30	15.7
	**Total**	**191**	**100.0**
**WHO Stage of HIV**			
	Stage I	80	33.3
	Stage II	107	44.6
	Stage III	45	18.8
	Stage IV	8	3.3
	**Total**	**385**	**100.0**
**CD_4_^+^ T cell category**			
	<200 cells/µl	70	40.0
	200-499 cells/µl	68	38.9
	≥500 cells/µl	37	21.1
	**Total**	**175**	**100.0**
**ART Regimen**			
	Tenofovir containing regimen	374	97.1
	Zidovudine containing regimen	11	2.9
	**Total**	**385**	**100.0**

**Table 2:** Characteristics of community members assessed for their role in retention in HIV care at the Muyuka Health District, 2018

Characteristic		Frequency	Percentage (%)
Gender			
	Male	135	38.8
	Female	213	61.2
	Total	348	100.0
Age group (years)			
	21-30	191	54.9
	31-40	85	24.4
	41-50	42	12.1
	>50	30	8.6
	Total	348	100.0
Estimated monthly income level			
	Low level (1-100,000 CFA)	105	30.2
	Middle income (101,000-300,000 CFA)	118	33.9
	High income (> 301, 000CFA)	8	2.3
	None income (0 CFA)	117	33.6
	Total	348	100.0
Marital status			
	Single	179	51.4
	Married	147	42.2
	Divorced	13	3.7
	Co-habitation	9	2.6
Religion			
	Christianity	318	91.4
	Islamism	10	2.9
	Others	20	5.7
			
	Total	348	100.0

**Table 3:** Association of pre-retention characteristic and retention of patients enrolled in HIV care between January, 2016 and April, 2018 at the Muyuka District Hospital

Characteristic	Not retained No (%)	Retained No (%)	Odds ratio	95% CI	P-value
**ART Regimen**					
Tenofovir Containing Regimen	270 (72.2)	104 (27.8)	1.00	-	-
Zidovudine Containing Regimen	3 (27.3)	8 (72.7)	6.92	1.80-26.60	0.005

## Discussion

This study was conducted to determine community participation in the retention of adults in HIV care in the Muyuka Health District. In this study, the overall retention rate (29.1 %) was different from that described in a systemic review of patient retention in ART programs in sub-Saharan Africa (60%) [10, 11]. The findings from this study were also different from a study conducted in South Africa which showed that, lower health facilities were quite effective managing patients once task shifting, mentoring and community support for the patients were made an integral part of the scale up of HIV care [12]. On the other hand, the low retention rate of this study was similar to a study conducted in Uganda where patients enrolled in HIV care had a retention rate of 48%, because patients in this study were not routinely followed-up at the level of the community [13]. Possible explanations of the poor retention in HIV care in the MHD could be due to financial constraints, patient feeling better after registering some improvements, poor social support from partners and family members, fear of being stigmatized and low community-based follow-up of patients accessing care at the MHD, and the ongoing socio-political crises in the South West region of Cameroon. Loss to follow-up in this study within two years and four months was high (70.9%). This was different from findings of the ART-link Collaboration that analysed 18 HIV cohorts across developing countries which reported a 15% loss to follow-up in the first year [14]. Possible reasons leading to loss to follow-up in this study could be due to financial constraints limiting repeated travel to the unit, myths about ART, competition with traditional medicine, lack of disclosure, lack of home based support and fears of losing one's social status. There was a statistically significant association between ART regimen and appointment adherence. Patients on a Zidovudine containing ART regimen were more likely to be loss to follow-up compared to patients on a Tenofovir containing ART regimen. This could be due to the fact that Zidovudine has more serious side effects than Tenofovir and could lead to lack of interest to continue treatment on the part of the patient [15]. There was no statistically significant association between gender, age groups, weight, CD4 lymphocyte count and WHO staging of HIV patient. The overall community participation was low. Although there are not many readily available publications providing adequate evidence supporting our findings from this research, a few of the available literature shows an unambiguous positive impact of community support on a wide range of aspects, including access and coverage, adherence, virological and immunological outcomes, patient retention and survival [12, 16]. The evidence suggests that a durable programme of universal access to ART will not only require a new level of performance of the regular health system, but also the mobilisation of additional human resources namely: the community as a whole and community care givers in particular [17, 18]. In addition, the review demonstrates that community support initiatives are effective strategies to address the current HIV/AIDS epidemic. Given the pressure on health systems and their professional staff, the existing evidence suggests that programmes on community health workers, although not necessarily cheap or easy, remain a good investment to improve coverage of communities with much needed health services, such as ART [13, 19].

## Conclusion

The retention rate of patients enrolled in HIV care was low (29.1%) as well as the level of community participation in the retention of adults in HIV care. We recommend that community members should participate more in activities that enhance the retention of patients in HIV care such as the creation of community based organisations and becoming HIV activists. Collaborations between the Ministry of Health and Community-Based Organizations (CBO_S_) should be encouraged to ensure continuum of care for the HIV/AIDS patients.

### What is known about this topic

- Very little has been published on community participation in the retention of adults in HIV care;
- Available literature suggests that community involvement towards the care of HIV/AIDS patients will have a positive impact on the survival of HIV/AIDS patients.

### What this study adds

- Retention status of people living with HIV (PLWHIV) in the Muyuka Health District is low (29.1%);
- Community involvement towards the care of people living with HIV (PLWHIV) in the Muyuka Health District is low;
- Enhancing community participation towards the care of people living with HIV (PLWHIV) would increase coverage and access to health care in order to achieve viral load suppression among HIV/AIDS patients.

## Competing interests

The authors declare no competing interests.
